# Association of *MMP-2*-753C>T and *MMP-9*-1562C>T Polymorphisms with Chronic/Aggressive Periodontitis Risk: A Systematic Review and Meta-Analysis

**Published:** 2019-07

**Authors:** Fatemeh MASHHADIABBAS, Hossein NEAMATZADEH, Elnaz FOROUGHI, Seyed Alireza DASTGHEIB, Soudabeh FARAHNAK, Rezvan NASIRI, Shima AHMADI

**Affiliations:** 1. Department of Maxillofacial Pathology, School of Dentistry, Shahid Beheshti University of Medical Sciences, Tehran, Iran; 2. Department of Medical Genetics, School of Medicine, Shahid Sadoughi University of Medical Sciences, Yazd, Iran; 3. Mother and Newborn Health Research Center, School of Medicine, Shahid Sadoughi University of Medical Sciences, Yazd, Iran; 4. Department of Pediatric Dentistry, School of Dentistry, Arak University of Medical Sciences, Arak, Iran; 5. Department of Medical Genetics, School of Medicine, Shiraz University of Medical Sciences, Shiraz, Iran; 6. Department of Endodontics, School of Dentistry, Arak University of Medical Sciences, Arak, Iran; 7. Department of Prosthodontics, School of Dentistry, Shahid Sadoughi University of Medical Sciences, Yazd, Iran

**Keywords:** Periodontitis, MMP gene, Polymorphism, Meta-analysis

## Abstract

**Background:**

Two functional polymorphisms in the matrix metalloproteinase-2 and -9 (*MMP-2* and *MMP-9*) genes may contribute to periodontitis pathogenesis. However, the results were inconsistent and inconclusive. Therefore, to clarify precise associations of *MMP-2*-753 C>T and *MMP-9*-1562C>T polymorphisms with chronic (CP) and aggressive (AgP) periodontitis, we performed a systematic review and meta-analysis.

**Methods:**

A literature search was conducted using PubMed, Google Scholar, Embase, and Web of Science databases until 5 July 2017. The data were analyzed with CMA software, and risk estimates are expressed as odds ratios (ORs) and 95% confidence intervals (95% CIs).

**Results:**

Nineteen case-control studies in ten publications with 2089 periodontitis cases and 2345 controls met the criteria. The pooled ORs indicated that *MMP-2*-753C>T and *MMP-9*-1562C>T polymorphisms were not significantly associated with risk of periodontitis in overall analysis. Stratified analyses by ethnicity and periodontitis type indicated that the *MMP-9*-1562C>T polymorphism showed a significant association with the risk of periodontitis among Caucasians and CP/AgP subgroup, whereas *MMP-2*-753C>T polymorphism was significantly associated with periodontitis risk only among Asians.

**Conclusion:**

*MMP-2*-753C>T and *MMP-9*-1562C>T polymorphisms may not be associated with risk of periodontitis in overall population. However, *MMP-2*-753C>T and *MMP-9*-1562C>T polymorphisms might have influence on the susceptibility of periodontitis by ethnicity.

## Introduction

Periodontitis is one of the most common causes of inflammatory bone loss in human (1–3). Periodontitis is a complex multifactorial disease that involves the interaction of environmental factors such as smoking and the patient’s related factors such as sex, age and systemic diseases (1,4). Majority of the population has experienced some level of gingival inflammation worldwide, and 5%–8% of the population suffering from severe forms of periodontitis (5). Both chronic and aggressive forms of periodontitis are characterized by inflammation (6). Periodontitis as most human diseases have a genetic component, which influences inflammatory and immune responses in this disease (7). Results from the twin and family studies, indicate a role of genetic component in development periodontitis (8,9). Many studies have shown a correlation between periodontitis and systemic disease involving genes such as Matrix Metalloproteinases (MMP) as a shared mechanism of inflammation (8,10,11).

Matrix metalloproteinases constitute a family of 25 zinc-dependent proteolytic enzymes, which are capable of degrading the extracellular matrix (ECM) (10–12). *MMP-2* (Gelatinase-A) and -9 (Gelatinase-B) are two widely studied matrix metalloproteinases (13). *MMP-2* gene (also known as 72-kDa type IV collagenase) is located on human chromosome 16q12.2 (14). *MMP-2* gene encodes a protein that involved in the breakdown of ECM in normal physiological processes, such as embryonic development, reproduction and tissue remodeling (15). *MMP-9* gene (also known as 92-kDa gelatinase or type V collagenase) is located on human chromosome 20q11.2–q13.1 (16). *MMP-9* encodes a multidomain enzyme, a class of enzymes that belong to the zinc-metalloproteinases family involved in the degradation of the ECM (16,17). In the past decade, several epidemiologic studies have been investigated the association of *MMP-9* -1562C>T (rs3918242) and *MMP-2* -753C>T (rs2285053) polymorphisms with susceptibility to periodontitis (8,10,11). However, those studies results remain fairly inconsistent and inconclusive. A meta-analysis is a very powerful tool to obtain sufficient statistical power to detect the potential effect of these polymorphisms from individual studies with small size and the statistically low power.

Thus, we performed the current systematic and meta-analysis of all available case-control studies to provide more precise estimation of the association of *MMP-2* -753C>T (rs2285053) and *MMP-9* -1562C>T (rs3918242) polymorphisms with chronic/aggressive periodontitis susceptibility.

## Materials and Methods

### Search Strategy

A systematic search of eligible studies on the association of *MMP-2* -753C>T (rs2285053) and *MMP-9* -1562C>T (rs3918242) polymorphisms with periodontitis susceptibility was conducted in PubMed, ISI Web of Science, Google Scholar, and Embase databases up to July 15, 2017. The search strategies were based on combinations of the following keywords: (‘‘Matrix Metallopeptidase or ‘‘collagenase’’ or ‘’MMP’’ or ‘’*MMP*-2’’ or ‘’gelatinase A’’ or ‘*’MMP-9’’* or ‘’gelatinase B’’ or ‘*’MMP-9* -1562C>T’’ or ‘*’MMP-2* -753C>T‘’ or ‘’ rs3918242’’ or ‘’ rs2285053’’) and (“periodontitis’’ or “periodontal disease” or ‘’chronic periodontitis’’ or ‘’CP’’ ‘’aggressive periodontitis’’ or ‘’AgP’’) and (‘‘gene’’ or ‘‘allele’’ or ‘‘genotype’’ or ‘‘mutation’’ or ‘‘variant’’ or ‘’single nucleotide polymorphisms’’ or ‘’SNPs’’ or ‘‘variation’’ or ‘‘polymorphism’’). The extracted publications were limited to English. Additionally, we have screened the references list of the retrieved original articles for more additional original articles. If there were multiple reports of the same study or overlapping data only the study with the largest sample sizes or the most recent one was included to the present meta-analysis.

### Inclusion and Exclusion Criteria

Studies were included based on the following criteria: 1) only full-text and published studies; 2) studies with case-control or cohort design; 3) a study evaluated the association of *MMP-9* -1562C>T (rs3918242) and *MMP-2* -753C>T (rs2285053) polymorphisms with periodontitis (CP and/or AgP) susceptibility risk; 4) available genotypes frequencies of *MMP-9* -1562C>T (rs3918242) and *MMP-2* -753C>T (rs2285053) polymorphisms were provided to estimate the odds ratios (ORs) with 95% confidence intervals (CIs). The exclusion criteria were as follows: 1) the study was not conducted on periodontitis; 2) abstracts, case reports, and review articles; 3) studies with only case group (no control group); 4) studies on the other polymorphisms of the *MMP-2* and *MMP-9* genes; 5) studies without detail genotype frequencies, which were unable to calculate ORs; and 6) duplicate publications of data from the same study.

### Data Extraction

Two authors independently extracted the following data from each eligible study according to the inclusion criteria: the first author’s name, the year of publication, ethnicity, country of origin, total number of cases and controls, the frequencies of genotypes, minor allele frequencies (MAFs), and Hardy-Weinberg equilibrium test in control subjects. We have calculated the allele frequencies from corresponding genotype distributions using an online website. Any discrepancy between these two authors was resolved by reaching a consensus through discussion or the involvement of a third author who made the final decision through discussions.

### Statistical Analysis

Odds ratio (OR) and 95% confidence intervals (CIs) were calculated to evaluate the strength of the associations of *MMP-9* -1562C>T and *MMP-2* -753C>T polymorphisms with risk of periodontitis. The significance of the pooled OR was determined by the Z-test. Pooled ORs were performed for both *MMP-9* -1562C>T and *MMP-2* -753C>T polymorphisms under the allele model (T vs. C), the heterozygote model (TC vs. CC), the homozygote model (TT vs. CC), the dominant model (TT+TC vs. CC), and the recessive model (TT vs. TC+CC). Heterogeneity (between-study inconsistency) was assessed by both the chi-square-based Q statistic (considered significant with *P*<0.10) and the *I^2^* statistic. In the current meta-analysis the *I^2^* values of 25, 50, and 75% meant a low, moderate, and high heterogeneity, respectively. When heterogeneity existed (*P*<0.10), a random-effects model weighted by the DerSimonian–Laird method was used to give a more conservative result; otherwise, a fixed-effects model weighted by the Mantel–Haenszel method would be applied. Sensitivity analyses were performed by omitting each particular study at a time. Hardy–Weinberg equilibrium (HWE) was evaluated for each study by Chi-square test in control groups, and *P*<0.05 was considered as a significant departure from HWE. Moreover, sensitivity analysis was also performed, excluding studies whose allele frequencies in controls exhibited significant deviation from the Hardy–Weinberg equilibrium (HWE), given that the deviation may denote bias. A meta-regression analysis was carried out to identify the major sources of between-studies variation in the results, using the log of the ORs from each study as ethnicity and types of periodontitis as the possible sources of heterogeneity. Moreover, the quality of selected studies was tested by the confirmation of HWE in control groups, and studies without the confirmation of HWE in controls were defined as low-quality studies, while studies with the confirmation of HWE in controls were defined as high-quality studies. Funnel plots and Egger’s test were used to examine publication bias (*P*<0.05). If publication bias existed, the Duval and Tweedie non-parametric ‘‘trim and fill’’ method was used to adjust for it. The statistical analysis for the current meta-analysis study was performed using the Comprehensive Meta-Analysis (CMA) software (version 2.2; Biostat, USA). In the current meta-analysis, all *P*-values were considered two-sided, and *P*=0.05 was set as the threshold value for statistical significance.

## Results

### Characteristics of the Included Studies

**Fig. 1:**
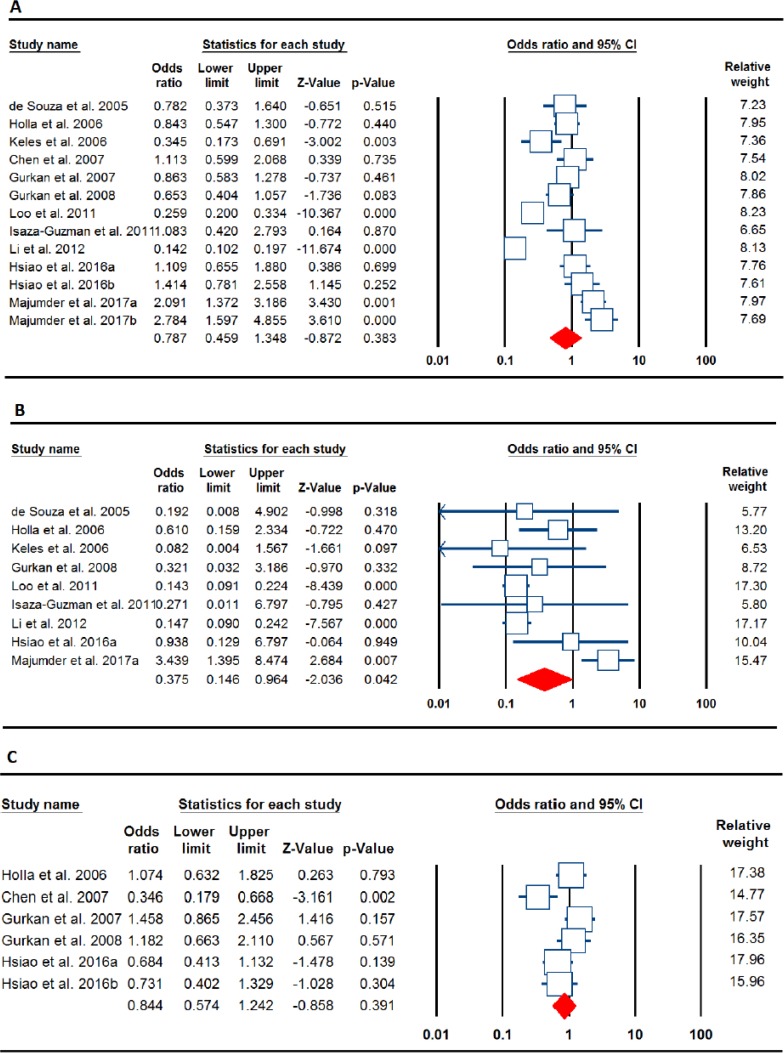
Forest plots for the association of the *MMP-2* -753C>T and *MMP-9* -1562C>T polymorphisms with periodontitis risk. **A:** *MMP-9* -1562C>T (allele model: T vs. C), **B:** *MMP-9* -1562C>T (CP, homozygote model: TT vs. CC), and **C:** *MMP-2* -753C>T (dominant model: TT+TC vs. CC)

As shown in Fig. 1, the comprehensive search of literature under defined terms retrieved 197 articles. Of those 185 articles were excluded through duplicate screening and screening of titles and abstracts. Next, 45 studies were excluded because were reviews, case reports, irrelevance to the topic, not involving periodontitis research and lacking sufficient data. Finally, 19 case-control studies from ten publications (18–28) were identified in the meta-analysis. Basic characteristics of the selected articles are all listed in Table 1.

**Table 1:** Main characteristics of studies included in this meta-analysis

***First Author***	***Country (Ethnicity)***	***Periodontitis Type***	***Case/Control***	***Cases***					***Controls***					***MAFs***	***HWE***
				***Genotypes***			***Allele***		***Genotypes***			***Allele***			
***MMP-9 -1562C>T***				***CC***	***CT***	***TT***	***C***	***T***	***CC***	***CT***	***TT***	***C***	***T***		
de Souza 2005 ^18^	Brazil(Mixed)	CP	62/38	42	20	0	104	20	24	13	1	61	15	0.1974	0.623
Holla 2006 ^19^	Czech(Caucasian)	CP	169/135	122	43	4	287	51	93	37	5	223	47	0.1741	0.586
Keles 2006 ^20^	Turkey(Caucasian)	CP	70/70	57	13	0	127	13	42	24	4	108	32	0.2286	0.586
Chen 2007 ^21^	China(Asian)	AgP	79/128	62	15	2	139	19	101	26	1	228	28	0.1094	0.629
Gurkan 2007 ^22^	Turkey(Caucasian)	AgP	112/157	58	53	1	169	55	78	72	7	228	86	0.2739	≤0.001
Gurkan 2008 ^23^	Turkey(Caucasian)	CP	87/107	54	32	1	140	34	52	52	3	156	58	0.271	0.017
Loo. 2011 ^24^	China(Asian)	CP	280/250	143	73	64	359	201	43	72	135	158	342	0.684	0.001
Isaza-Guzman 2011 ^25^	Colombia(Mixed)	CP	69/54	58	11	0	127	11	47	6	1	100	8	0.0741	0.163
Li 2012 ^26^	China(Asian)	CP	122/532	68	26	28	162	54	99	156	277	354	710	0.6673	0.001
Hsiao 2016a ^27^	Taiwan(Asian)	CP	129/117	96	31	2	223	35	90	28	2	205	29	0.1333	0.916
Hsiao 2016b ^27^	Taiwan(Asian)	AgP	69/117	48	19	2	115	23							
Majumder 2017a ^28^	India(Asian)	CP	110/121	53	39	18	145	75	81	32	8	194	48	0.1983	0.063
Majumder. 2017b ^28^	India(Asian)	AgP	38/121	17	11	10	45	31							
*MMP-2 -753C>T*				CC	CT	TT	C	T	CC	CT	TT	C	T		
Holla 2006 ^19^	Czech(Caucasian)	CP	149/127	107	38	4	255	43	93	30	4	216	38	0.149	0.419
Chen 2007 ^21^	China(Asian)	AgP	167/128	63	15	1	298	36	98	28	2	224	32	0.125	1.000
Gurkan. 2007 ^22^	Turkey(Caucasian)	AgP	92/157	49	39	4	137	47	98	54	5	250	64	0.203	0.454
Gurkan 2008 ^23^	Turkey(Caucasian)	CP	87/107	51	32	4	134	40	67	37	3	171	43	0.200	0.426
Hsiao 2016a ^27^	Taiwan(Asian)	CP	129/117	75	44	10	194	64	57	48	12	162	72	0.307	0.688
Hsiao 2016b ^27^	Taiwan(Asian)	AgP	69/117	39	26	4	104	34	57	48	12				

CP: Chronic periodontitis; AgP: Aggressive periodontitis; Minor allelic frequency; HWE, Hardy-Weinberg equilibrium.

All studies were case-control in design. Of these case-control studies, for the *MMP-9* -1562C>T polymorphism, 13 case-control studies in ten publications (18–28) were available, including 1396 cases and 1709 controls. For the *MMP-2* -753C>T polymorphism, six case-control studies (19,21–23,27) involved a total of 693 cases and 636 controls. Among the 13 eligible studies for *MMP-9* -1562C>T (rs3918242), four case-control studies (19,20,22,23) including 438 cases and 469 controls were undertaken in Caucasians (Czech and Turkey), seven case-control studies (21,24,26–28) containing 827 cases and 1144 controls were conducted in Asians (China, Taiwan, and India), and two case-control studies (18,25) with 131 cases and 92 controls was performed in Latinos populations (Brazil and Colombia). Of six case-control studies for MMP-2-753C>T (rs2285053), three case-control studies (19,22,23) including 328 cases and 391 controls were undertaken in Caucasians (Czech and Turkey) and three case-control studies (21,27) with 365 cases and 245 controls were performed in Asian (China and Taiwan) populations. The control populations of four studies deviated from Hardy–Weinberg equilibrium (HWE). Moreover, the minor allele frequencies (MAFs) for controls and genotype distributions for *MMP-9* -1562C>T (rs3918242) and *MMP-2* -753C>T (rs2285053) polymorphisms in different ethnicities are all listed in Table 1.

### Quantitative Synthesis

#### MMP-9 -1562C>T Polymorphism

**Fig. 2:**
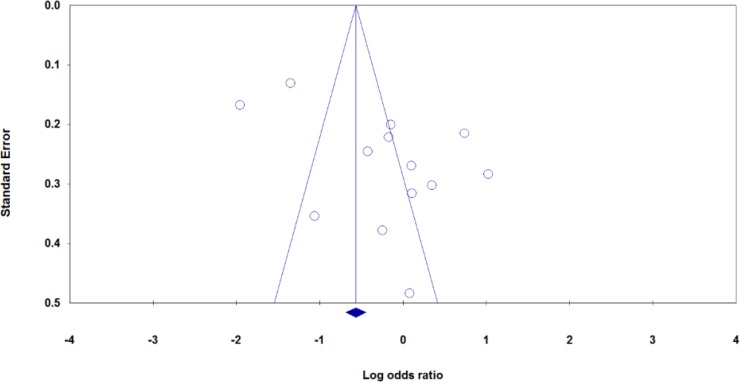
Begg’s funnel plots of the *MMP-9*-1562C>T polymorphism and periodontitis risk for publication bias test under allele model (T vs. C)

The main results of *MMP-9* -1562C>T polymorphism meta-analysis are shown in Table 2. The pooled results based on all included studies not showed a significant association between *MMP-9* -1562C>T and periodontitis risk under all genetic models (allele model: T vs. C, OR=0.787, 95% C=0.459–1.348, *P*=0.383 (Fig. 2A); heterozygote model: TC vs. CC, OR=0.795, 95% CI=0.546–1.156, *P*=0.229; homozygote model: TT vs. CC, OR=0.600, 95% CI=0.237–1.517, *P*=0.280 (Fig. 2B); dominant model: TT+TC vs. CC, OR=0.767, 95% CI=0.463–1.269, *P*=0.301; and recessive model: TT vs. TC+CC, OR=0.691, 95% CI=0.330–1.444, *P*=0.326). In the subgroup analyses by the disease type, there was a significant association between *MMP-9* -1562C>T polymorphism and periodontitis risk under the homozygote model (TT vs. CC: OR=0.375, 95% C=0.146–0.964, *P*=0.042) and the recessive model (TT vs. TC+CC: OR=0.473, 95% C=0.235–0.954, *P*=0.036) in the CP group. In addition, there was a significant association between *MMP-9* -1562C>T polymorphism and periodontitis under the recessive model (TT vs. TC+CC: OR=2.585, 95% C=1.177–5.678, *P*=0.018) in the AgP group. In the subgroup analyses by ethnicity, there was a significant association between *MMP-9* -1562C>T polymorphism and periodontitis risk under all genetic models (allele model: T vs. C, OR=0.723, 95% C=0.572–0.914, *P*=0.007; heterozygote model: TC vs. CC, OR=0.753, 95% CI=0.568–1.000, *P*=0.050; homozygote model: TT vs. CC, OR=0.347, 95% CI 0.133–0.909, *P*=0.031; dominant model: TT+TC vs. CC, OR=0.709, 95% CI=0.538–0.936, *P*=0.015; and recessive model: TT vs. TC+CC, OR=0.378, 95% CI=0.145–0.983, *P*=0.046) in the Caucasians, but not in Asian and Latinos populations. Moreover, when stratifying the studies by HWE status, a significant association between *MMP-9* -1562C>T polymorphism and periodontitis risk was observed only under recessive model (TT vs. TC+CC, OR=1.873, 95% CI=1.123–3.125, *P*=0.016) (Table 2).

**Table 2:** The results of meta-analysis for association of *MMP-9* -1562C>T polymorphism with periodontitis risk

***Polymorphism***	***Genetic Model***	***Type of Model***	***Heterogeneity***		***Odds Ratio***				***Publication Bias***	
			***I^2^(%)***	***P_H_***	***OR***	***95% CI***	***Z_test_***	***P_OR_***	***P_Beggs_***	***P_Eggers_***
Overall	T vs. C	Random	94.15	≤0.001	0.787	0.459–1.348	−0.872	0.383	0.854	0.033
	TC vs. CC	Random	78.19	≤0.001	0.795	0.546–1.156	−1.202	0.229	0.427	0.180
	TT vs. CC	Random	86.17	≤0.001	0.600	0.237–1.517	−1.080	0.280	0.854	0.156
	TT+TC vs. CC	Random	89.69	≤0.001	0.767	0.463–1.269	−1.034	0.301	0.582	0.050
	TT vs. TC+CC	Random	79.48	≤0.001	0.691	0.330–1.444	−0.983	0.326	0.854	0.206
Periodontitis Type										
CP	T vs. C	Random	94.45	≤0.001	0.611	0.321–1.163	−1.500	0.134	0.754	0.140
	TC vs. CC	Random	81.95	≤0.001	0.676	0.416–1.101	−1.573	0.116	0.465	0.326
	TT vs. CC	Random	82.78	≤0.001	0.375	0.146–0.964	−2.036	0.042	0.916	0.357
	TT+TC vs. CC	Random	90.85	≤0.001	0.607	0.324–1.137	−1.558	0.119	0.602	0.175
	TT vs. TC+CC	Random	71.17	0.001	0.473	0.235–0.954	−2.092	0.036	1.000	0.452
AgP	T vs. C	Random	74.32	0.009	1.373	0.812–2.320	1.183	0.237	1.000	0.405
	TC vs. CC	Fixed	0.00	0.716	1.112	0.806–1.534	0.644	0.519	0.308	0.283
	TT vs. CC	Random	63.47	0.042	1.849	0.407–8.396	0.796	0.426	0.734	0.307
	TT+TC vs. CC	Fixed	41.65	0.162	1.199	0.880–1.632	1.151	0.250	0.308	0.238
	TT vs. TC+CC	Fixed	60.89	0.053	2.585	1.177–5.678	2.367	0.018	0.734	0.327
By ethnicity										
Caucasian	T vs. C	Fixed	48.15	0.122	0.723	0.572–0.914	−2.717	0.007	0.089	0.013
	TC vs. CC	Fixed	36.74	0.192	0.753	0.568–1.000	−1.962	0.050	0.089	0.044
	TT vs. CC	Fixed	0.00	0.594	0.347	0.133–0.909	−2.155	0.031	0.308	0.058
	TT+TC vs. CC	Fixed	46.13	0.135	0.709	0.538–0.936	−2.429	0.015	0.089	0.015
	TT vs. TC+CC	Fixed	0.00	0.640	0.378	0.145–0.983	−1.994	0.046	0.308	0.107
Asian	T vs. C	Random	96.84	≤0.001	0.856	0.345–2.121	−0.336	0.737	0.763	0.035
	TC vs. CC	Random	87.48	≤0.001	0.812	0.424–1.555	−0.628	0.530	0.367	0.073
	TT vs. CC	Random	92.92	≤0.001	1.017	0.279–3.710	0.026	0.979	0.229	0.067
	TT+TC vs. CC	Random	94.36	≤0.001	0.806	0.339–1.919	−0.487	0.626	0.367	0.015
	TT vs. TC+CC	Random	89.37	≤0.001	1.092	0.404–2.946	0.173	0.863	0.229	0.061
Mixed	T vs. C	Fixed	0.00	0.596	0.885	0.494–1.585	−0.412	0.681	NA	NA
	TC vs. CC	Fixed	0.00	0.453	1.081	0.553–2.111	0.228	0.820	NA	NA
	TT vs. CC	Fixed	0.00	0.883	0.228	0.023–2.242	−1.267	0.205	NA	NA
	TT+TC vs. CC	Fixed	0.00	0.512	0.978	0.509–1.879	−0.066	0.947	NA	NA
	TT vs. TC+CC	Fixed	0.00	0.915	0.227	0.023–2.213	−1.277	0.202	NA	NA
By HWE										
	T vs. C	Random	75.10	≤0.001	1.138	0.771–1.679	0.652	0.514	0.602	0.337
	TC vs. CC	Fixed	33.17	0.152	1.067	0.848–1.342	0.554	0.579	0.916	0.921
	TT vs. CC	Random	53.28	0.029	1.366	0.570–3.273	0.699	0.485	0.175	0.026
	TT+TC vs. CC	Random	63.18	0.005	1.102	0.761–1.597	0.516	0.606	0.916	0.696
	TT vs. TC+CC	Fixed	45.56	0.065	1.873	1.123–3.125	2.402	0.016	0.175	0.028

CP: Chronic periodontitis; AgP: Aggressive periodontitis; NA: not applicable

### MMP-2-753C>T Polymorphism

The main results of *MMP-2* -753C>T polymorphism meta-analysis are shown in Table 3. The overall analyses suggested no significant association between the *MMP-2* -753C>T polymorphism and periodontitis susceptibility in all genetic models (allele model: T vs. C, OR=0.940, 95% C=0.780–1.132, *P*=0.513; heterozygote model: TC vs. CC, OR=0.985, 95% CI=0.776–1.249, *P*=0.898; homozygote model: TT vs. CC, OR=0.827, 95% CI=0.486–1.406, *P*=0.482; dominant model: TT+TC vs. CC, OR=0.844, 95% CI=0.574–1.242, *P*=0.391 (Fig. 2C); and recessive model: TT vs. TC+CC, OR=0.828, 95% CI=0.492–1.394, *P*=0.477). In the subgroup analyses, there was not a significant association between *MMP-2* -753C>T polymorphism and periodontitis risk under all five genetic models in the CP and AgP groups. We then performed stratified analysis by ethnicity and found a significant association between the *MMP-2* -753C>T polymorphism and periodontitis risk in the Asians under the allele model (T vs. C, OR=0.766, 95% C=0.590–0.996, *P*=0.046) and the recessive mode (TT vs. TC+CC, OR=0.587, 95% CI=0.320–1.237, *P*=0.002), but not in Caucasian populations.

**Table 3:** The results of meta-analysis for association of *MMP-2* -753C>T polymorphism with periodontitis risk

***Polymorphism***	***Genetic Model***	***Type of Model***	***Heterogeneity***		***Odds Ratio***			***Publication Bias***		
			***I^2^(%)***	***P_H_***	***OR***	***95% CI***	***Z_test_***	***P_OR_***	***P_Beggs_***	***P_Eggers_***
Overall	T vs. C	Fixed	17.36	0.301	0.940	0.780–1.132	−0.654	0.513	1.000	0.832
	TC vs. CC	Fixed	0.00	0.463	0.985	0.776–1.249	−0.128	0.898	1.000	0.571
	TT vs. CC	Fixed	0.00	0.715	0.827	0.486–1.406	−0.703	0.482	0.707	0.384
	TT+TC vs. CC	Random	64.75	0.014	0.844	0.574–1.242	−0.858	0.391	0.452	0.212
	TT vs. TC+CC	Fixed	0.00	0.797	0.828	0.492–1.394	−0.711	0.477	0.707	0.899
Periodontitis Type CP	T vs. C	Fixed	9.48	0.331	0.914	0.706–1.182	−0.689	0.491	0.296	0.192
	TC vs. CC	Fixed	0.00	0.386	0.940	0.680–1.299	−0.375	0.708	0.296	0.440
	TT vs. CC	Fixed	0.00	0.536	0.834	0.421–1.652	−0.522	0.602	0.296	0.306
	TT+TC vs. CC	Fixed	15.62	0.306	0.931	0.684–1.269	−0.451	0.652	0.296	0.330
	TT vs. TC+CC	Fixed	0.00	0.657	0.890	0.455–1.739	−0.341	0.733	0.296	0.403
AgP	T vs. C	Fixed	46.58	0.154	0.969	0.740–1.270	−0.225	0.822	1.000	0.372
	TC vs. CC	Fixed	21.62	0.279	1.040	0.731–1.480	0.219	0.826	1.000	0.314
	TT vs. CC	Fixed	0.00	0.438	0.816	0.351–1.898	−0.472	0.637	1.000	0.898
	TT+TC vs. CC	Random	82.45	0.003	0.731	0.324–1.650	−0.754	0.451	0.296	0.059
	TT vs. TC+CC	Fixed	0.00	0.494	0.741	0.323–1.697	−0.709	0.478	1.000	0.805
By ethnicity Caucasian	T vs. C	Fixed	0.00	0.587	1.162	0.890–1.516	1.104	0.269	1.000	0.551
	TC vs. CC	Fixed	0.00	0.753	1.227	0.888–1.692	1.238	0.216	1.000	0.549
	TT vs. CC	Fixed	0.00	0.763	1.333	0.583–3.046	0.681	0.496	1.000	0.823
	TT+TC vs. CC	Fixed	0.00	0.713	1.233	0.902–1.686	1.311	0.190	1.000	0.768
	TT vs. TC+CC	Fixed	0.00	0.798	1.237	0.546–2.805	0.510	0.610	1.000	0.766
Asian	T vs. C	Fixed	0.00	0.906	0.766	0.590–0.996	−1.998	0.046	0.296	0.536
	TC vs. CC	Fixed	0.00	0.912	0.759	0.533–1.079	−1.537	0.124	0.296	0.108
	TT vs. CC	Fixed	0.00	0.918	0.590	0.295–1.181	−1.489	0.136	1.000	0.834
	TT+TC vs. CC	Fixed	40.21	0.188	0.587	0.421–0.818	−3.144	0.002	1.000	0.488
	TT vs. TC+CC	Fixed	0.00	0.836	0.630	0.320–1.237	−1.343	0.179	0.296	0.274

CP: Chronic periodontitis; AgP: Aggressive periodontitis.

### Minor Allele Frequencies (MAFs)

The allele and genotype distributions of *MMP-9* -1562C>T and *MMP-2* -753C>T polymorphisms by ethnicity are presented in Table 1. The minor allele frequencies of the *MMP-9* -1562C>T and *MMP-2* -753C>T polymorphisms exhibited ethnic variations. The *MMP-9* -1562T allele frequencies in the Asians, Caucasians and Latinos populations were 39.65% (10.9%–68.4%), 22.35% (17.4%–27.3%) and 13.55% (7.4%–19.7%), respectively. The *MMP-2* -753T allele frequencies in the Asians and Caucasians were 21.6% (12.5%–30.7%) and 17.6% (14.9%–20.3%), respectively. Therefore, the frequencies of the *MMP-9* -1562T and *MMP-2* -753T alleles in Caucasians were less than Asians.

### Sensitivity Analysis

We have performed sensitivity analysis by omitting individual studies to assess the effect of each publication on the overall results. However, the significance of pooled ORs not influenced by omitting those studies, indicating that the results were stable. Additionally, we have performed sensitivity analysis by omitting six studies in which the genotype distributions of *MMP-9* -1562C>T polymorphism in the healthy controls significantly deviated from the HWE. The results showed a significant association between *MMP-9* -1562C>T polymorphism and periodontitis risk under recessive model (TT vs. TC+CC, OR=1.873, 95% CI=1.123–3.125, *P*=0.016), which suggests that the results of our meta-analysis are affected by HWE status.

### Publication Bias

We have performed Begg’s funnel plot and Egger’s test to detect the publication bias of included studies. The shape of the funnel plot did not reveal any evidence of obvious asymmetry for *MMP-2* -753C>T and *MMP-9* -1562C>T polymorphisms under all genetic models. However, the results of Egger’s test showed evidence of publication bias for *MMP-9* -1562C>T under allele model (T vs. C: P_Begg’s_=0.854, P_Egger’s_=0.033). Then, we have used the Duval and Tweedie non-parametric ‘‘trim and fill’’ method to adjust the publication bias. However, meta-analysis with and without ‘‘trim and fill’’ did not draw different conclusion, indicating that our findings were statistically robust.

## Discussion

In general, several studies revealed the relation between *MMP-2* -753C>T and *MMP-9* -1562C>T polymorphisms and susceptibility of periodontitis, however; the main findings from the different case-control studies did not reach the same conclusion. This inconsistency may result from the small sample size and the different experimental methods such as genotyping methods, ethnicity background, and subject’s gender. The present study showed that the *MMP-2* -753C>T and *MMP-9* -1562C>T polymorphisms were not associated with the susceptibility of periodontitis in overall analysis. However, there is still a need for further research and screening of etiological relations of the *MMP-2* and 9 genes functional polymorphisms with the susceptibility of periodontitis. The limited statistical results may be reasonable due to the differences in the ethnic background, because different populations have different frequencies of alleles, and different genetic backgrounds may affect periodontitis risk. Therefore, we have performed subgroup analyses by ethnicity and disease type. The stratified analyses by ethnicity and periodontitis type revealed that the *MMP-9* -1562C>T polymorphism showed a significant association with the risk of periodontitis among Caucasians and CP/AgP subgroup, whereas *MMP-2* -753C>T polymorphism was significantly associated with periodontitis risk only among Asians.

We found that the presence of T allele in *MMP-2* -753C>T and *MMP-9* -1562C>T polymorphisms is not significantly associated with an increased risk of periodontitis. Recently, two meta-analyses estimated the association between *MMP-2* -753C>T and *MMP-*9 -1562C>T polymorphisms and periodontitis risk, which was basically in accordance with our results that *MMP-2* -753C>T and *MMP-9* -1562C>T polymorphisms may not contribute to the susceptibility of periodontitis in overall analysis (8,11). Moreover, at least four case-control studies (in two publications) (27,28) have not included in the meta-analysis (8). Additionally, Li et al. have included only two studies for *MMP-2* -753C>T and *MMP-9* -1562C>T polymorphisms (11). Thus, the ongoing uncertainty still exists and the conclusion by might be biased by not inclusion of new published studies (8,11).

In our meta-analysis, we accurately assessed the association between these *MMP-2* -753C>T and *MMP-9* -1562C>T polymorphisms and the risk of chronic/aggressive periodontitis by taking into account the effects of new published studies. Moreover, a significant association had not found between *MMP-2* -753C>T polymorphism and periodontitis according to disease type and ethnicity (8), while we have found that *MMP-2* -753C>T polymorphism was significantly associated with periodontitis risk only among Asians. Therefore, both *MMP-2* -753C>T and *MMP-9* -1562C>T polymorphisms might have influence on the susceptibility of periodontitis by ethnicity background. Additionally, in the present meta-analysis we have provided the actually numbers of minor allele frequencies (MAFs) in the controls. The conclusion by our meta-analysis was more credible.

Between-study heterogeneity is very common and expected in the genetic association studies of meta-analysis (29,30). Therefore, it is necessary to evaluate the magnitude of heterogeneity in a meta-analysis for determining the strengths of pooled results (31,32). We found relatively a high heterogeneity (>70%) for *MMP-9* -1562C>T in overall analysis in all genetic models, but not for *MMP-2* -753C>T polymorphism. We suggested that several factors including genetic backgrounds for cases and controls, diverse genotype distribution of *MMP-2* -753C>T and *MMP-9* -1562C>T polymorphisms in the included ethnicity groups, types of periodontitis (CP/AgP), different genotyping methods, sample size of included studies, and uneven selection criteria for the cases and controls in different studies, responsible for such heterogeneity in our meta-analysis. Moreover, we have performed subgroup analysis by ethnicity, periodontitis type and HWE status to finding source of heterogeneity. The heterogeneity was reduced in the AgP group and also disappeared in Caucasians and Latinos populations, but not in the Asians and by HWE status. In addition, we have found that that the heterogeneity was significantly reduced in the small sample size group in all genetic models, suggesting that the total sample size, ethnicity background, periodontitis type and HWE status were the sources of heterogeneity.

Although we performed a comprehensive meta-analysis, some limitations should be acknowledged. First, the number of published studies for *MMP-2* -753C>T polymorphism was not sufficiently large for a comprehensive analysis, especially for stratified analyses by ethnicity. Second, the majority of the included studies was Asians or Caucasians, because of limited available data for *MMP-2* -753C>T and *MMP-9* -1562C>T polymorphisms from another ethnicity such as Africans, our results should be interpreted with caution. Larger studies are needed to clarify whether these two polymorphisms could truly affect the risk of periodontitis in other ethnicities. Third, our results were based on single-factor estimates without adjustment for other risk factors, therefore; our results may be affected by additional confounding factors, such as age, gender, smoking status, another chronic disease such as diabetes, caused serious confounding bias. If we had been able to acquire more detailed data, we would have achieved estimations that are more precise by adjusting for other potential covariates, but most of the included studies either did not report the data or aggregated them in different ways, making it impossible to include the data in the current meta-analysis. Therefore, studies with good design are needed in the future, and ORs adjusted for other confounding factors need reporting. Finally, it is also possible that language bias will exist, as in the present meta-analysis we have only included articles published in English. Finally, we have not addressed the effect of gene-gene and gene-environment interactions.

## Conclusion

*MMP-2* -753C>T and *MMP-9* -1562C>T polymorphisms may not be associated with periodontitis risk in overall analysis. However, both *MMP-2* -753C>T and *MMP-9* -1562C>T polymorphisms might have influence on the susceptibility of periodontitis by ethnicity background.

## Ethical considerations

Ethical issues (Including plagiarism, informed consent, misconduct, data fabrication and/or falsification, double publication and/or submission, redundancy, etc.) have been completely observed by the authors.
